# Current Understanding of Exosomal MicroRNAs in Glioma Immune Regulation and Therapeutic Responses

**DOI:** 10.3389/fimmu.2021.813747

**Published:** 2022-01-14

**Authors:** Jinwu Peng, Qiuju Liang, Zhijie Xu, Yuan Cai, Bi Peng, Jianbo Li, Wenqin Zhang, Fanhua Kang, Qianhui Hong, Yuanliang Yan, Mingyu Zhang

**Affiliations:** ^1^ Department of Pathology, Xiangya Hospital, Central South University, Changsha, China; ^2^ Department of Pathology, Xiangya Changde Hospital, Changde, China; ^3^ Department of Pharmacy, Xiangya Hospital, Central South University, Changsha, China; ^4^ National Clinical Research Center for Geriatric Disorders, Xiangya Hospital, Central South University, Changsha, China; ^5^ Department of Neurosurgery, Xiangya Hospital, Central South University, Changsha, China

**Keywords:** exosomes, microRNAs, glioma, biomarker, therapeutic response

## Abstract

Exosomes, the small extracellular vesicles, are released by multiple cell types, including tumor cells, and represent a novel avenue for intercellular communication *via* transferring diverse biomolecules. Recently, microRNAs (miRNAs) were demonstrated to be enclosed in exosomes and therefore was protected from degradation. Such exosomal miRNAs can be transmitted to recipient cells where they could regulate multiple cancer-associated biological processes. Accumulative evidence suggests that exosomal miRNAs serve essential roles in modifying the glioma immune microenvironment and potentially affecting the malignant behaviors and therapeutic responses. As exosomal miRNAs are detectable in almost all kinds of biofluids and correlated with clinicopathological characteristics of glioma, they might be served as promising biomarkers for gliomas. We reviewed the novel findings regarding the biological functions of exosomal miRNAs during glioma pathogenesis and immune regulation. Furthermore, we elaborated on their potential clinical applications as biomarkers in glioma diagnosis, prognosis and treatment response prediction. Finally, we summarized the accessible databases that can be employed for exosome-associated miRNAs identification and functional exploration of cancers, including glioma.

## Introduction

Malignant gliomas, representing 80% of the whole primary brain tumors, are the most prevalent and fatal primary neoplasm of the central nervous system in adults (1). Clinicopathologically, glioma is categorized into grades I–IV based on the histologic criteria proposed by the World Health Organization (WHO), with grade IV, glioblastoma (GBM), as the most malignant (2–4). Despite the enhanced understanding of the molecular mechanism of gliomas and considerable progress in therapeutic approaches encompassing surgery, radiotherapy, chemotherapy and targeted therapy, recurrence is still observed in nearly all malignant gliomas, which generally causes death (5, 6). Patients suffering from gliomas show an unsatisfactory prognosis, with a median survival time of approximately 12-15 months after diagnosis (7). Apart from the rapid proliferation, high aggressiveness, genetic heterogeneity and therapeutic recalcitrance of glioma, the poor survival of glioma patients also results from the insufficient understanding of the specific molecular mechanisms controlling disease progression and shortage of reliable tools for timely diagnosis and sensitive therapeutic monitoring (8). Hence, the molecular mechanisms correlated with glioma development and progression remain unclear, so do the non-invasive biomarkers with high sensitivity and specificity.

Extracellular vesicles (EVs), membrane-encased vesicles secreted by cells, deliver cytoplasmic or membrane contents to nearby cells and are detectable in biological fluids (9, 10). Although EVs were initially relegated as entities for cellular waste disposal, nowadays, they work as messengers in intercellular communication (11). EVs are either buds from the endosomal network (exosomes) or derived from the plasma membrane (microvesicles) (10). Exosomes are lipid bilayer-encapsulated vesicles with a diameter ranging from 30 to 120 nm and are shed by diverse cell populations containing neoplastic cells. With multiple biologically active cargoes, including proteins, lipids, mRNAs, and microRNAs (miRNAs), exosomes have emerged as important players in cell-to-cell communication (12). Exosomes are generated in endosomal compartments termed multivesicular bodies (MVBs) that are late endosomes encompassing various intraluminal vesicles (ILVs) formed by endosomal membrane invaginations. MVBs can subsequently integrate with plasma membranes, leading to the release of exosomes (13). Subsequently, exosomes are internalized into neighboring or distant cells and transport their components, thereby influencing the phenotype of target cells (14). Mounting evidence reveals that exosome-mediated intercellular communication also serves important roles in many respects of cancer progression, covering metastasis and drug resistance as well as interfering with the immune systems within the tumor microenvironment (15, 16). It was reported that tumor cells produce high levels of exosomes, where the components vary in distinct pathological and physiological conditions (17). Recent data confirmed that circulating exosomes appear as a promising means for biomarker discovery since they can be noninvasively collected, and their *in vivo* half-life is short, and their cargoes are protected from degradation (18).

MiRNAs is a kind of small non-coding RNAs, with 19-25 nucleotides that form base pairs with the 3’-untranslated regions (3’-UTR) of target mRNAs, which further cause either mRNA destabilization or translational repression (19, 20). MiRNAs function as irreplaceable intercellular communication tools, as they are transmitted between cells through exosomes and impact recipient cells’ phenotype (21). Exosomal miRNAs have been connected with glioma progression *via* activation and/or suppression of certain signaling pathways (22). A better knowledge of the biological roles of exosomal miRNAs may facilitate the exploration and development of novel diagnoses and therapies for gliomas. In this review, we outlined the current findings regarding exosomal miRNAs involvement in tumor initiation and progression, emphasizing glioma cancer. Moreover, we summarized the available tools and platforms that help investigate the underlying regulatory mechanisms of exosomal miRNAs in gliomas. Finally, we highlighted the potential value of exosomal miRNAs in the future clinical application of gliomas.

## Proposed Roles of Exosomal miRNAs in Glioma Cancer

**Figure 1:**
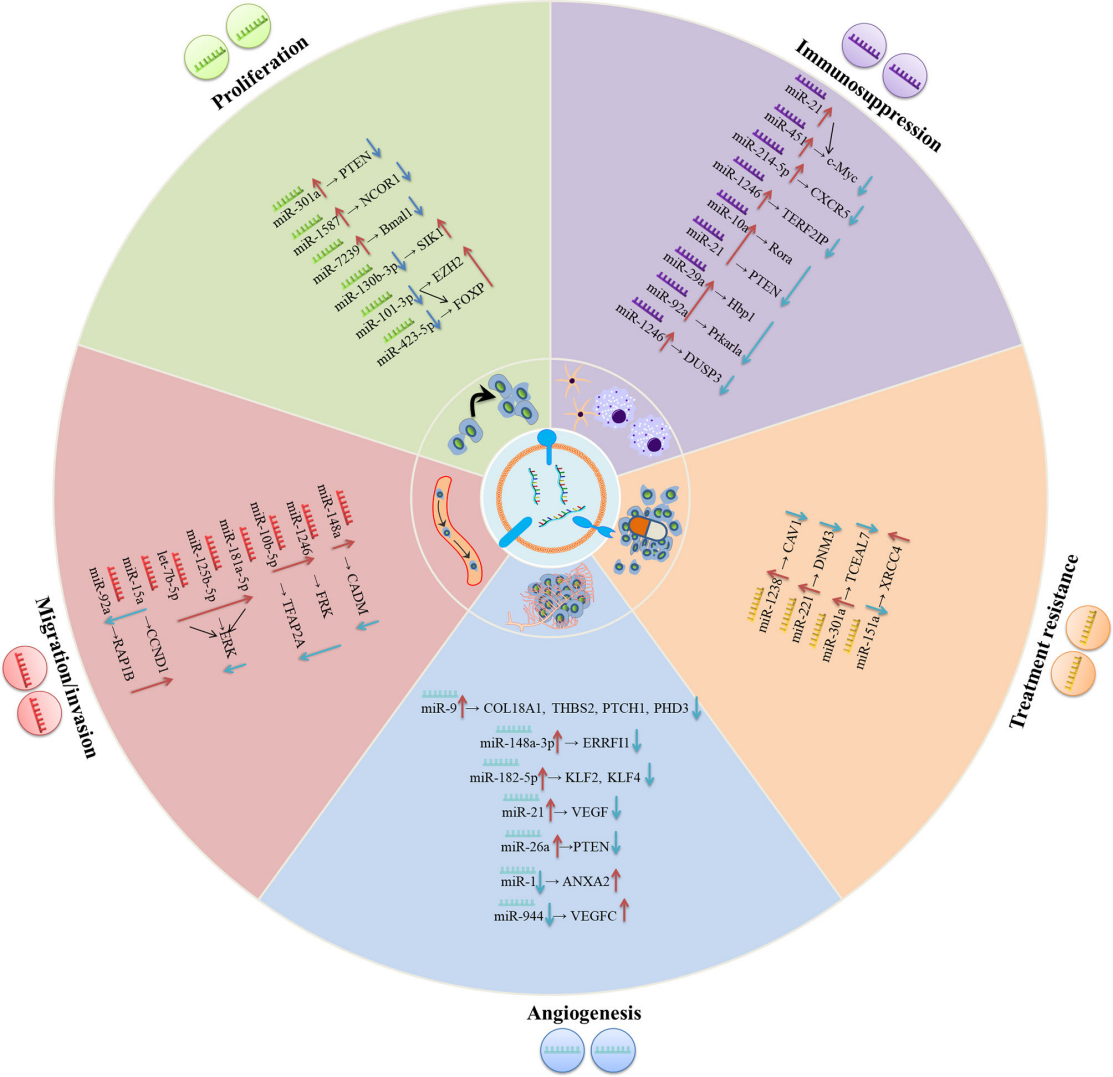
The underlying molecular mechanisms of exosomal miRNAs in modulating the progression of gliomas. In the glioma environment, exosomal miRNAs are absorbed by recipient cells and subsequently exert their functions in varieties of biological processes including proliferation, migration/invasion, angiogenesis, treatment resistance, immunosuppression, etc.

Accumulative evidence reveals that exosomes mediate the initiation and progression of gliomas by transferring biomolecules between distinct cell populations (23). Among these molecules, exosomal miRNAs are the most intriguing due to their important role in multi-respect glioma biology, encompassing proliferation, migration and invasion, angiogenesis, immune suppression, and treatment resistance within the glioma microenvironment (**Table 1**, **Figure 1**). Notably, the effects of exosomal miRNAs on glioma are highly similar to non-exosomal-derived miRNAs.

**Table 1:** The biological roles of exosomal miRNAs in glioma biology.

Exosomal miRNAs	Donor cells/recipient cells	Targets	Biological functions	References
miR-301a	GBM^i^ cells/low grade H4 glioma cells	PTEN	Promoting proliferation and invasion	(24)
miR-1587	GA-hMSCs^ii^/GSCs^iii^	NCOR1	Promoting proliferation	(25)
miR-7239-3p	M2 microglial/glioma cells	Bmal1	Promoting proliferation and invasion	(26)
miR-130b-3p	mono-macrophages/MB^iv^ cells	SIK1	Suppressing proliferation, migration and invasion	(27)
miR-101-3p	mono-macrophages/MB cells	FOXP4 (common target)	Suppressing proliferation, migration and invasion	(28)
miR-423-5p		EZH2 (target gene of miR-101-3p)	Promoting apoptosis	
miR-148a	GBM cells /GBM cells	CADM1	Promoting proliferation and metastasis	(29)
miR-1246	hypoxic glioma cells/normoxic glioma cells	FRK	Promoting migration and invasion	(30)
miR-10b-5p		TFAP2A		
miR-181a-5p, miR-125b-5p, let-7b-5p	Group 3 MB cells/ SHH MB cells	ERK	Promoting invasion and migration	(31)
miR-15a	M2 macrophage/glioma	CCND1	Suppressing migration and invasion	(32)
miR-92a		RAP1B		
miR-1	GBM cells/HBMVEC^v^	ANXA2	Suppressing angiogenesis and invasion	(33)
miR-9	glioma cells / HUVECs^vi^	COL18A1, THBS2, PTCH1, PHD3	Promoting proliferation, migration, invasion and angiogenesis	(34)
miR-148a-3p	glioma cells/HUVECs	ERRFI1	Promoting angiogenesis and proliferation	(35)
miR-182-5p	GBM cells/HUVECs	KLF2 and KLF4	Promoting angiogenesis	(36)
miR-21	GSC/HBMVEC	VEGF	Promoting angiogenesis	(37)
miR-26a	GSCs/HBMECs	PTEN	Promoting proliferation, migration and angiogenesis	(38)
miR-944	GSCs/HUVECs	VEGFC	Suppressed proliferation, migration and angiogenesis	(39)
miR-1238	TMZ^vii^-resistant cells/TMZ-sensitive cells	CAV1	Promoting resistance to TMZ	(40)
			Enhancing anti-apoptosis	
miR-151a	TMZ-resistant GBM cells/TMZ-sensitive GBM cells	XRCC4	Increasing chemosensitivity to TMZ	(41)
miR-221	glioma cells/glioma cells	DNM3	Promoting proliferation, migration and TMZ resistance	(42)
miR-301a	hypoxia glioma cells/normaxia-cultured glioma cells	TCEAL7	Promoting radiation resistance	(43)
miR-451	glioma cells/microglia or macrophages	c-Myc	Promoting proliferation and immunosuppression	(44)
miR-214-5p	GBM Cells/ microglia cells	CXCR5	Creating a tumor-supportive milieu	(45)
miR-1246	hypoxia glioma cells /macrophages	TERF2IP	Promoting immunosuppression	(46)
			Promoting proliferation and metastasis	
miR-10a	hypoxic glioma cells/MDSC^viii^	Rora/IκBα/NF-κB,	Promoting immunosuppression	(47)
miR-21		Pten/PI3K/AKT pathways		
miR-29a	hypoxia glioma cells /MDSC	Hbp1	Promoting immunosuppression and proliferation	(48)
mir-92a		Prkar1a		
miR-1246	(hypoxia) glioma cells/PBMCs^ix^	DUSP3	Promoting immunosuppression	(49)

i Glioblastoma.

^ii^glioma related-mesenchymal stem cells.

^iii^glioma stem-like cells.

^iv^medulloblastoma.

human brain microvascular endothelial cells.

^vi^human umbilical vein endothelial cells.

^vii^Temozolomide.

^viii^myeloid-derived suppressor cells.

^ix^peripheral blood mononuclear cells.

### Exosomal miRNAs and Proliferation of Glioma

Proliferation is an important part of cancer progression, characterized by the alteration of expression and/or activity of cell cycle-associated proteins. Cell growth is also stimulated by constitutively activated signal transduction pathways (50). Exosomes shuttle genetic messages between cells *via* exosomal miRNAs within the tumor environment, thus contributing to glioma cell proliferation. Exosomal miRNAs regulate the proliferation of glioma cells in the following ways. Glioma cells-derived exosomes are transmitted to glioma cancer cells, and exosomal miRNAs modulate the proliferation of recipient cells. For instance, Lan et al. observed the significantly enhanced levels of exosomal miR-301a in serum of glioma patients relative to healthy individuals. Moreover, miR-301a delivered by exosomes derived from GBM cells promoted proliferation and invasion of low-grade H4 glioma cells through directly targeting phosphatase and tensin homolog (PTEN) to enhance the AKT and FAK signaling pathways (24). Non-glioma-derived exosomes are transmitted to glioma cells and further modulate their proliferation. Figueroa et al. found that miR-1587 could be transferred by glioma related-mesenchymal stem cells (GA-hMSCs) to glioma stem-like cells (GSCs) *via* exosomes and increased GSC proliferation and clonogenicity to maintain a GSC-supportive niche *via* directly targeting the expression of nuclear receptor co-repressor-1 (NCOR1) (25). Li et al. also revealed that miR-7239-3p, released by M2 microglial exosomes, could enter glioma cells *via* endocytosis, resulting in the repression of brain and muscle ARNT-like protein-1 (Bmal1) expression and facilitating glioma cells proliferation and migration (26). In addition, the molecule mechanisms of exosomal miRNA in medulloblastoma (MB), another subtype of glioma, have been recently investigated. Based on the finding of Huang’s group, exosomal miR-130b-3p activated the p53 signaling pathway *via* silencing serine/threonine-protein kinase 1 (SIK1), thereby suppressing the proliferation, migration and invasion of MB cells (27). In line with this report, Xue et al. demonstrated that exosomes derived from MB patients’ plasma could shuttle miR-101-3p and miR-423-5p to MB cells and suppress the proliferation, invasion and migration of MB cells. Mechanically, miR-101-3p and miR-423-5p exert their suppressive effect *via* a common target, forkhead box P4 (FOXP4), while miR-101-3p also binds to the 3’-UTR of enhancer of zeste homolog 2 (EZH2) to reinforce its inhibitory effects on tumors (28). In short, these studies indicated that selectively transferring miRNAs *via* exosomes between cells represents an essential means for intercellular communication, and for modulating the proliferation of gliomas.

### Exosomal miRNAs and Invasion/Migration of Glioma

It is generally believed that the invasion of cancerous cells into surrounding vasculatures and tissues is the initial step for cancer metastasis, a leading cause of cancer-associated death (51). Malignant gliomas have a unique invasion capacity: impeding the surgical removal of all glioma cells and making relapse inevitable (52, 53). Accumulative evidence suggests that exosomal cargos containing nucleic acids such as miRNAs play decisive roles in glioma migration and invasion. Cai et al. found that exosomal miR-148a expression was prominently elevated in the serum of GBM patients and was inversely related to the expression of cell adhesion molecule 1 (CADM1). MiR-148a delivered by glioma-derived exosomes could target CADM1 and then activate STAT3 pathway to facilitate the proliferation and metastasis of GBM cells (29). Numerous studies have illustrated that hypoxia leads to the epithelial-to-mesenchymal transition (EMT) (54), tumor invasion (55) and metastasis (56). Qian et al. found that the hypoxia microenvironment could stimulate miR-1246 in glioma. Besides, miR-10b-5p-enriched exosomes were then internalized by normoxia glioma cells to enhance the migration and invasion of glioma through suppressing fyn-related kinase (FPK) and Transcription factor AP-2 alpha (TFAP2A), respectively (30). Similarly, exosomal miRNAs could also drive the migration and invasion of MB cells. Transfer of Group 3 MB-derived exosomal miRNAs (miR-181a-5p/miR-125b-5p/let-7b-5p) could promote the invasion and migration of less invasive SHH MB cells by enhancing extracellular regulated kinases (ERK) activities in Ras/MAPK pathway (31). Finally, the exosomes released by cancer cells and the exosomes secreted from other cell types participated in glioma invasion and migration. MiR-15a and miR-92a were poorly expressed in M2 macrophages exosomes. It has been verified that M2 macrophages could secret miR-15a and miR-92a *via* exosomes, and subsequently, miR-15a and miR-92a separately repressed cyclin D1 (CCND1) and Ras-related protein Rap1b (Rap1b). Thus, the PI3K/AKT/mTOR signaling pathway was interfered, and glioma migration and invasion was suppressed (32).

### Exosomal miRNAs and Angiogenesis in Glioma

As a pivotal hallmark of tumors, angiogenesis is a requisite for cancers to satisfy their needs for nutrients and oxygen (57). Aberrant angiogenesis could lead to malignant phenotypes and promote cancer metastasis (58). It is also established that angiogenesis relates to the progression of glioma (59). Nevertheless, the molecular mechanisms that regulate glioma angiogenesis are still unclear and will be the focus of current research. Since Folkman first discovered a theory about the correlation between angiogenesis and tumor growth, anti-angiogenic gene therapy has attracted the attention of many scientists with its apparent advantages (60). The development of angiogenesis inhibitors targeting pro-angiogenic signaling pathways regulated by vascular endothelial growth factor (VEGF) has exhibited clinical benefits in the treatment of varieties of cancers. While the resistance of patients to anti-VEGF therapy impeded cancer treatment (61). Therefore, a better understanding of the molecular mechanisms of tumor angiogenesis is necessary for a more effective anti-Angiogenic therapy.

The critical roles of miRNA in regulating tumor angiogenesis (62) were highlighted in studies. Exosomes that selectively packed miRNA and shed from tumor cells can be internalized by endothelial cells (ECs), thus facilitating the growth of new blood vessels (63, 64). Increasing evidence has revealed that exosomes regulate angiogenesis partially through delivering miRNAs in the glioma microenvironment. MiR-1 is a well-recognized tumor suppressor in several cancers (65). Loading miR-1 into GBM-derived EV could alleviate angiogenesis, invasion, and neurosphere formation of GBM cells through directly targeting and inhibiting annexin A2 (ANXA2). ANXA2 was evidenced to be an important oncogene whose expression was inversely correlated with miR-1 in GBM cells (33). In addition, miR-9 derived from glioma cells was internalized by vascular endothelial cells, which enhanced angiogenesis. Mechanically, miR-9 could target collagen type XVIII alpha 1 chain (COL18A1), thrombospondin 2 (THBS2), patched 1 (PTCH1), and egl-9 family hypoxia-inducible factor 3 (PHD3) to degrade these mRNA, contributing to initiating HIF-1α/VEGF signaling transduction (34). Besides, glioma-derived exosomal miR-148a-3p reinforced angiogenesis through activating the EGFR/MAPK signaling pathway with the ERBB receptor feedback inhibitor 1 (ERRFI1) (35).

Hypoxia is a universal manifestation of solid tumors. It is caused by the rapid tumor growth that runs out the supply of oxygen, and impairment of blood flow results from the aberrant blood vessels in the tumor (66). It can promote angiogenesis and tumor progression *via* altering the tumor microenvironment (TME) (67, 68). In a recent study by Li et al., hypoxic GBM-secreted miR-182-5p could be taken up by human umbilical vein ECs (HUVECs) *via* exosomes, which could promote angiogenesis by directly suppressing Kruppel-like Factor 2 and 4 (KLF2 and KLF4) and subsequently elevating VEGF receptors (VEGFR) expression (36).

Glioma stem cells (GSCs) are tumor cells that exist in primary GBM with stem-cell-like properties such as self-renew capacity and producing heterogeneous offspring (69). Moreover, GSCs have been elucidated to participate in tumor growth and angiogenesis (70, 71). GSCs trigger tumor angiogenesis by releasing factors that facilitate ECs proliferation and tube formation (72). Existing evidence demonstrated that GSCs secrete extracellular vesicles (containing exosomes) that include pro-angiogenic proteins, mRNA and miRNA, which are absorbed by human brain microvascular ECs (HBMVECs) (37–39, 73, 74). Sun et al. identified that overexpressed miR-21 in GSCs-derived exosomes could activate the angiogenic capacity of ECs by augmenting levels of VEGF, which further interplayed with VEGFR2 to trigger downstream PI3-kinase/Akt pathway (37). Furthermore, Wang et al. showed that GSCs-derived exosomes carrying miR-26a promoted the tube formation of HBMECs *in vitro* by targeting and suppressing PTEN, which activated the PI3K/Akt signaling pathway (38). Finally, a recently published report confirmed that exosome-mediated diffusion of miR-944 from GSCs to HUVECs decreased proliferation and angiogenesis by decreasing VEGFC expression and remarkably restraining Akt/ERK pathways (39). Thus, GSCs play a dual role in regulating angiogenesis in ECs, which depends on the exosomal miRNA.

### Exosomal miRNAs and Treatment Resistance of Glioma

The mainstay treatments against glioma remain surgical resection, which is followed by chemotherapy and radiotherapy. Although these methods can remove the majority of the tumor masses, tumor recurrence remains inevitable, which raises great challenges to clinical management owing to treatment resistance (75). Evidence revealed that GSCs with intrinsic resistance to therapy cause the recurrence (76). Exosomes secreted by both tumor and stromal cells play an integral role in treating resistance because of their nature as mediators of intercellular communication (77). Moreover, it has been suggested that the key exosomal miRNA could target diverse signaling pathways or affect regulatory proteins and their corresponding genes, thereupon modulating GBM drug resistance (78). Exploring the machinery of treatment resistance is relevant for eliminating these aggressive tumors.

Temozolomide (TMZ), as a DNA alkylating agent, is the first-line treatment for GBM (79) and has been confirmed to exert its anti-tumor role through inducing DNA damage (80). TMZ treatment significantly prolongs the survival of GBM patients, while the existence of resistance limited the clinic efficacy (81). Relevant studies indicated that exosomes derived from TMZ-resistant glioma could confer TMZ chemoresistance to the recipient TMZ-sensitive cells *via* exosomes (40, 41). Thus, drug resistance increased with the secretion of exosomes (82). Yin et al. reported that bioactive miR-1238 could be incorporated into TMZ-resistant GBM cells-derived exosomes and absorbed by TMZ-sensitive cells, which disseminated TMZ resistance by directly targeting and suppressing caveolin-1 (CAV1) and subsequently activating EGFR-PI3K-Akt-mTOR pathway. The epidermal growth factor receptor (EGFR) played a pivotal role in TMZ resistance (40). Additionally, Zeng et al. demonstrated that miR-151a showed downregulated expression in TMZ-resistant GBM cells and recurrent specimens and was correlated with TMZ resistance. Further study revealed that TMZ-resistant GBM cells spread TMZ chemoresistance to TMZ-responsive GBM cells under an exosomal miR-151a loss-dependent condition. Mechanically, X-ray cross-complementing gene 4 (XRCC4) was directly targeted by miR-151a, and the repression of miR-151a elevated XRCC4 levels, activating DNA repair and increasing the resistance of glioma to TMZ (41). Finally, the level of serum exosomal miR-221 increased with the glioma grades, and gliomas with higher grades displayed a higher level of miR-221. Exosomal miR-221 was connected with decreased TMZ sensitivity by targeting dynamin-3 (DNM3) genes (42).

Exosomal miRNAs shed by glioma cells are also associated with the sensitivity to radiotherapy, which can impact treatment efficiency. According to recent data, exosomal miR-301a, which was specifically expressed and released by hypoxia GBM cells, could transfer to corresponding normoxia-cultured cells, where it repressed the expression of anti-oncogene transcription elongation factor A (SII)-like 7 (TCEAL7) and subsequently activated the Wnt/β-catenin Signaling pathway, thus decreasing radiation sensitivity (43).

### Exosomal miRNAs and Immunosuppression in Glioma

Glioma-related immune cells, the substantial component in the glioma microenvironment, play an emerging role in the regulation of cancer progression and control of anti-tumor immunity (83–85). Macrophages/microglia (GAMs) and tumor-induced myeloid-derived suppressor cells (MDSCs), two prominent populations within the tumour stroma, can exert pro-tumorigenic effects and establish an immunosuppressive milieu (86, 87). M2 phenotypic conversion in glioma-associated GAMs and MDSCs expansion can help neoplastic cells evade immune system-mediate detection and destruction (88). Emerging studies suggest that exosomal miRNAs are carriers of information that have the potential to modulate macrophage fate of differentiation (46) and induce MDSCs accumulation and expansion (48), thereupon creating a favorable environment for glioma progression. Therefore, targeting these immune cells and relevant molecules appears as a novel and promising therapy for gliomas.

GAMs occupy the largest proportion in tumor-infiltrating cells for glioma and take up 30% of the whole glioma mass (89). The appearance and intensity of GAMs are closely related to gliomas progression, and conclusive evidence reveals the indispensable role of interplay between GAMs and gliomas in creating an immunosuppressive milieu, consequently favoring glioma growth and invasion (90). Nevertheless, the specific functioning mechanisms of tumor-infiltrated GAMs are yet to be addressed. Based on the report by Van der Vos, GBM-derived EVs were absorbed into microglia and monocyte/macrophage *via in vivo* combined with *in vivo* methods. That resulted in the transfer of miR-21 and miR-451, two abundant miRNAs within GBM-EVs with known oncogenic properties, into the latter cell types and decreased the level of c-Myc mRNA, a shared target of both miRNAs. The absorption of GBM-EVs was accompanied by alterations in microglia phenotype, covering enhanced proliferation and a shift in their cytokines profile towards immunosuppression (44). Furthermore, Yang and his colleagues demonstrated that the expression of miR-214-5p was aberrantly enhanced in GBM cells. It could be transferred into recipient microglia through exosomes, leading to the repression of C-X-C motif chemokine receptor 5 (CXCR5) and ameliorating the expression of pro-inflammatory cytokines such as interleukin 6 (IL-6), interleukin 8 (IL-8) and tumor necrosis factor-α (TNF-α) to regulate the inflammatory response of microglial cells, which helps create a tumor-supportive milieu (45). Besides, tumor-associated macrophages (TAM) are one of the main immune-related cells infiltrating the tumor microenvironment. They are generally categorized into two subtypes with distinct functions, namely classically activated (M1 macrophages) and alternatively activated (M2 macrophages), respectively. The former is characterized by anti-tumor activities, while the latter increases metastasis of tumors and angiogenesis and suppresses the anti-tumor immune response (91). Tumors provide a tumor-permissive milieu by exploiting macrophage polarization states and specifically skewing macrophages toward a pro-tumoral M2-like phenotype, thereby supporting cancer progression by immune suppression (92). What’s more, emerging evidence indicates that exosomal miRNAs secreted by glioma cells can control the phenotypic plasticity of macrophages to facilitate tumor growth. For example, the recently published literature by Qian has determined that miR-1246 was upregulated in hypoxia glioma-derived exosomes (H-GDE) and GBM patients’ cerebrospinal fluid (CSF), and delivery of H-GDE-derived miR-1246 contributed to inducing M2 macrophage polarization through targeting telomeric repeat binding factor 2 interacting protein (TERF2IP). Thus, the STAT3 signaling pathway was activated and NF-κB signaling pathway was repressed, promoting the development of the immunosuppressive microenvironment (46).

Myeloid-derived suppressor cells (MDSCs), as a heterogeneous population of cells expanding during cancer, can depress activation of T-cells and NK-cells to promote tumor growth, quicken formation of the pre-metastatic niche, and cause resistance to immunotherapy (93). Previous studies have evidenced that tumor-derived exosomes drive MDSCs activation and expansion (94), and miRNAs play essential roles in regulating the expansion of functional MDSCs (95). However, the exact mechanisms where miRNA-containing exosomes derived from glioma cells can manipulate the differential of functional MDSCs remains to be defined. To the end, Guo et al. identified that hypoxia-stimulated glioma-derived exosomes (GDEs) could be taken up into MDSCs, exhibiting a stronger capacity to induce MDSCs compared with normoxia-stimulated GDEs. It was exactly the hypoxia-inducible enhanced expressed miR-10a and miR-21 within GDEs that reinforced MDSCs expansion and activation through targeting Rora/IκBα/NF-κB and Pten/PI3K/AKT pathways (47). Guo et al. also revealed that exosomal miR-29a and miR-92a, which were transferred from hypoxia-induced glioma cells to MDSCs could facilitate the formation of the immunosuppressive microenvironment by increasing the proliferation of functional MDSCs *via* silencing high-mobility group box transcription factor1 (Hbp1) and protein kinase cAMP-dependent type I regulatory subunit alpha (Prkar1a), separately (48). Additionally, they also demonstrated that miR-1246, which was abundant in GDEs, could also potentiate MDSCs with manifestations including repressing CD8+ cells proliferation and elevating the levels of interleukin 10 (IL-10) and transforming growth factor-beta (TGF-β) by activating the DUSP3/ERK pathway (49). The above-described studies indicate that glioma potently influences MDSCs differentiation and activation *via* exosomal miRNAs, thereupon impacting the entire tumor immune environment.

## Clinical Implications of Exosomal miRNAs in Glioma

### Exosomal miRNAs as Biomarkers for Glioma

Precise diagnosis, and the timely/comprehensive monitoring of therapeutic response, remain the main challenges in glioma patients’ care and treatment. Neuroimaging, magnetic resonance imaging (MRI) in particular, is regularly used for glioma diagnosis, staging, and monitoring therapeutic response. Although neuroimaging is suggestive of glioma diagnosis, there are still other brain lesions sharing radiological features, which makes differential diagnosis difficult (96). Additionally, the lowest resolution for the effective detection by MRI remains on the order of millimeters (97). Because of the dimension of a tumor cell, this disparity in scale translates into delayed diagnosis and treatment (98). Furthermore, MRI can exhibit the enhanced tumor volume following radiotherapy, generally, due to enhanced vascular permeability, an effect named pseudoprogression, and connected with the treatment (99). In the meantime, histopathologic examination of tumor specimens attained by surgery is currently recognized as the gold standard for glioma grading and typing. Nevertheless, this method bears high surgical risks, and repeated sampling of tumor specimens may be impractical (100). All these factors demonstrably highlight the urgent need for exploring minimally invasive biomarkers supportive for reliable and consistent diagnosis, prognostication, and treatment response prediction.

Although methods of early diagnosis and timely management of various cancer types have been established, such as detection of circulating tumor cells (CTCs) and cell-free nucleic acids (cfNAs) in the circulation, comparatively little progress has been made concerning clinical validation of intrinsic brain tumors. Broad sets of promising biomarkers have already been identified in the blood and CSF of patients afflicted with gliomas, but few were applied clinically (101).

In recent years, exosomes have become an emerging topic implicating multifaceted development and biological process through being secreted into nearly all human fluids, containing plasma, CSF, urine, breast milk, and saliva (102). Accumulative investigations have been centered on exosomes as they can be used as diagnostic, prognostic, and therapeutic markers (103). Previous studies have demonstrated that exosomes can cross the intact blood-brain barrier (BBB) (104). Besides, the bilayer-lipid membranes of exosomes protect the bioactive cargos they contain from enzymatic RNase degradation (105). MiRNAs within peripheral blood exosomes may be relevant biomarkers and therapeutic targets for glioma since they are tumor regulators possessing oncogene and suppressor gene roles (106). Exosomes are extractable from peripheral blood, and miRNAs can be detected by technologies. Exosomal miRNAs could be regarded as promising comprehensive biomarkers, as they possess the potential to present real-time information of disease and can predict progressive disease and assess the response of cancers to targeted therapies (106) (**Table 2**).

**Table 2:** Related studies about exosomal miRNAs from human fluids as diagnostic, prognostic and predictive biomarkers in glioma.

Exosomal miRNAs	Sample type	Expression status	Clinical value	Reference
miR-320	serum	↑	diagnostic biomarker for GBM^x^	(107)
miR-574-3p				
RUN6-1				
miR-182-5p	serum	↑	diagnostic biomarker for GBM	(108)
miR-328-3p		↓		
miR-339-5p		↓		
miR-340-5p		↓		
miR-485-3p		↓		
miR-486-5p		↑		
miR-543		↓		
miR-21	serum	↑	diagnostic biomarker for HGG^xi^	(109)
miR-222			diagnostic biomarke for grade prediction (HGG over LGG^xii^, miR-21)	
miR-124-3p				
miR-766-5p	serum	↓	diagnostic biomarker for discriminating HGG over intracranial lymphoma	(110)
		↓		
miR-301a	serum	↑	diagnostic biomarker for glioma	(24)
			prognostic biomarker for poor OS^xiii^	
miR-454-3p	serum	↑	diagnostic biomarker for glioma	(111)
			prognostic biomarker for poor survival	
miR-210	serum	↑	diagnostic biomarker for glioma and grade prediction (high grade over low grade)	(112)
			prognostic biomarker for recurrence and poor OS	
miR-181b	serum	↓	prognostic biomarker for shorter post-surgical survival time	(113)
miR-182-5p	serum	↑	prognostic biomarker for LGG	(114)
miR-223-3p		↑		
miR-34a-5p		↓		
miR-497-5p		↓		
miR-375	plasma	↑	diagnostic biomarker for glioma	(115)
miR-210	Plasma	↑	diagnostic and prognostic biomarker showing association with histopathological grade (GBM over LGA^xiv^)	(116)
miR-449		↓		
miR-5194		↓		
miR-2276-5p	Plasma	↓	diagnostic biomarker for glioma and grade prediction (HGG over LGG)	(117)
			Prognostic biomarker for better OS	
miR-21	CSF^xv^	↑	diagnostic biomarker for GBM	(118)
miR-21	CSF	↑	diagnostic biomarker for discriminating glioma patients over non-tumor brain disease	(119)
			diagnostic biomarker for grade prediction (GBM over grade II gliomas)	
			prognostic biomarker for poor survival	
miR-182-5p	Serum and CSF	↑	diagnostic biomarker for glioma and grade prediction (HGG over LGG)	(36)
			prognostic biomaker for glioma	
miR-1246	CSF	↑	prognostic biomarker for recurrence	(49)
miR-9-5p	Serum and CSF	↑	prognostic biomaker for poor survival	(120)
miR-151a	CSF	↓	predictive biomarker for TMZ^xvi^ response	(41)
miR-574-3p	serum	↑	predictive biomarker for the effect of radiotherapy for glioma patients	(121)
miR-21	Serum	↑	predictive biomarker for therapy response in post-surgical following-up	(122)
miR-222				
miR-124-3p				

x glioblastoma multiforme.

^xi^high-grade glioma.

^xii^low-grade glioma.

^xiii^overall survival.

^xiv^low-grade astrocytoma.

^xv^cerebrospinal fluid.

^xvi^Temozolomide.

Some studies have reported the diagnostic implication of exosomal miRNAs derived from sera. Exosomal miRNAs are correlated with histopathologic grades of gliomas. For instance, RNU6-1, miR-320, and miR-574-3p were fast and reliable diagnostic markers of GBM patients as their circulating levels increased with the area under the curve (AUC) of 0.926 (107). This research opened up a novel avenue that exosomal miRNAs could be applied for GBM diagnosis. Ebrahimkhani characterized a set of exosomal miRNAs (miR-182-5p, miR-328-3p, miR-339-5p, miR-340-5p, miR-485-3p, miR-486-5p as well as miR-543) derived from human serum to distinguish GBM patients from healthy controls with a predictive accuracy of 91.7% (108). Moreover, Santangelo validated that the expression of miR-21/miR-222/miR-124-3p in serum exosomes of patients with high-grade gliomas (HGG) was remarkably higher than those with low-grade gliomas (LGG) and healthy individuals. The combination of miR-21+miR-222+miR-124-3p was more robust in discriminating HGG than healthy controls, but its accuracy markedly decreased after surgical resection of the tumors. Intriguingly, exosome-associated miR-21 expression alone appeared as the greatest predictor for differentiating patients with HGG from those with LGG, with an AUC of 0.83. Meanwhile, the results also revealed that high exosomal miR-21 together with low exosomal miR-222 and miR-124-3p expression could effectively help to distinguish brain tumors of glial origin from those without glial origin at initial neuroradiological assessment, which may be helpful for patients showing inconclusive biopsies or with masses in the essential and intricate areas of the brain (109). Furthermore, according to the report from Wang’s group, both miR-766-5p and miR-376-5p in serum exosomes were strikingly attenuated in intracranial lymphoma, and HGG patients relative to healthy controls, and the levels of exosomal miR-766-5p were lower in the intracranial lymphoma group than the HGG group. Exosomal miR-766-5p and miR-376-5p could effectively diagnose HGG with AUCs of 0.8883 and 0.7688, respectively. Besides, miR-766-5p could be used as an auxiliary indicator for the diagnosis of HGG of intracranial lymphoma, showing an AUC value of 0.7201 (110). Additionally, exosomal miR-301a was significantly upregulated in the serum of glioma patients, which was connected with ascending pathological grades and decreased Karnofsky performance status (KPS) scores. ROC curve analysis revealed that exosomal miR-301a exhibited a potential diagnostic value for discriminating gliomas patients from non-glioma patients (AUC=0.937) (24). Research by Shao et al. revealed that miR-454-3p was markedly under-expressed in glioma tissues while over-expressed in exosomes, and exosomal miR-454-3p could function as a marker for glioma diagnosis with AUC value of 0.863. Levels of exosomal miR-454-3p in the postoperative serums were prominently lower relative to those in the preoperative serums (111). Finally, Lan et al. identified that exo-miR-210 whose expression was significantly reinforced in glioma patients and elevated with ascending pathological grades, could effectively identify glioma patients from healthy controls (AUC=0.856) (112).

The aforementioned dysregulations of exosomal miR-301a, miR-454-3p and miR-210 was also connected with the prognosis of glioma patients. Specifically, the expression of serum exosomal miR-301a was significantly diminished following the surgical removal of primary tumors and increased during GBM recurrence. Kaplan-Meier analysis suggested that malignant patients with an elevated exosomal miR-301a expression generally have a poorer survival (OS) (24). Similarly, enhanced expression of miR-454-3p (111) and miR-210 (112) in glioma serum exosomes were both related to poor prognosis. Another study also reported that augmented levels of exosomal miR-181b in GBM patients’ serum could indicate a worse functional outcome and suggest a prominently shorter post-surgical survival time for GBM patients (113). Also, Caponnetto et al. analyzed and compared the miRNA profile of exosomes released by glioma-associated stem cells (GASC), and confirmed that abnormal expression levels of exosomal miRNAs probably contributed to the malignant progression phenotypes. These exosomal miRNAs from serum warrant further investigation for the improvement of LGG prognostic stratification (114).

In addition to sera, increasing research have focused on identifying promising biomarkers based on exosomal miRNAs attained from glioma patients’ plasma. In a recent study by Xu et al, circulating exosomal miR-375 in the plasma of glioma patients was prominently reinforced and the extent of this upregulation was positively associated with tumor grades, indicating that miR-375-containing exosomes held great promise as a diagnostic marker (115). Similarly, Tabibkhooei et al. demonstrated that enhanced expression of miR-210 and declined expression of miR-5149 and miR-449 in plasma had a positive correlation with histopathological grade of glioma, which favored GBM detection and prognosis prediction. That implied that plasma exosomal miRNAs are expected to be novel biomarkers of GBM (116). Another study also confirmed that miR-2276-5p expression levels were declined in the plasma-derived exosomes of glioma patients compared with those of non-glioma controls and its expression was lower in HGG patients than in LGG patients. Exosomal miR-2276-5p could effectively diagnose glioma patients with an AUC of 0.8107. Furthermore, low levels of exosomal miR-2274-5p in glioma were linked to poorer survival rates (117).

Aberrant expression of exosomal miRNAs, a hallmark of glioma, can be detected in blood, and other body fluids, including CSF. For instance, Akers and colleagues proved that miR-21 expression levels were highly elevated in EVs derived from CSF of GBM patients relative to those from non-oncologic patients. CSF EV miR-21 could function as a feasible biomarker of GBM, with an AUC of 0.9 (118). Consistent with this finding, the data from Shi’s group have highlighted that according to its reinforced expression in gliomas, CSF-derived exosomal miR-21 appeared as an excellent index to distinguish glioma and non-neoplastic brain diseases yielding an AUC of 0.927. Moreover, it had the potential to specifically separate GBM and grade II gliomas with an AUC value of 0.751. The authors also identified that a high abundance of miR-21 bore an inverse correlation with patients’ survival (119). Both of the aforementioned studies suggest that expression levels of exosomal miR-21 in CSF could be a promising indicator applying for glioma diagnosis and prognosis. Another recently published paper has observed that glioma patients had enhanced expression of miR-182-5p from serum- and CSF-derived exosomes compared with healthy individuals, and high-grade patients, GBM patients, in particular, showcased higher exosomal miR-182-5p than LGG patients. Additionally, a dramatic drop in miR-182-5p expression was detected in the postoperative period. Exosome-mediated miR-182-5p, thereupon, could be a desirable diagnostic and prognostic biomarker of glioma (36). Moreover, CSF exosomal miR-1246 was associated with glioma and could potentially be applied for monitoring tumor recurrence after surgery (49). Finally, augmented expression of small EVs-derived miR-9-5p was linked to a decreased survival in IDH-mutated glioma patients, suggesting that miR-9-5p within the EVs from blood and CSF might be explored as a potential molecular biomarker (120).

Acquired drug resistance is emerging as a leading clinical limiting factor in the treatment of gliomas. Thereby, the identification of potential drug-resistant patients and exploration of alternative treatment timely are relevant to ameliorate prognosis. Exosomes can influence therapy resistance as delivery vehicles of miRNAs and can predict therapeutic responses as biomarkers. Zeng et al. identified that GBM patients with attenuated CSF exosomal miR-151a levels exhibited a worse prognosis, revealing a dismal response to TMZ (41). Additionally, miR-574-3p derived from serum exosomes was significantly diminished after radiotherapy, and it was proposed as an important candidate biomarker for evaluating the therapeutic effect of radiotherapy in glioma (121). Furthermore, reinforced expression levels of serum exosomal miR-21, miR-222 and miR-124-3p were associated with HGG progression. Specifically, after the chemo-radiotherapy, HGG patients with high levels of these miRNAs showed significantly shorter progression-free survival (PFS) and overall survival (OS) (122). Hence, the profiles of exosomal miRNAs could potentially predict the therapeutic response in glioma patients.

Exosomes contain not only miRNAs but also other noncoding RNAs (ncRNAs), including circular RNAs (circRNAs) or long non-coding RNAs (lncRNAs). Exosomal miRNA mediated glioma progression *via* interacting with circRNAs or lncRNAs. Accumulative studies have also focused on the expression of exosomal circRNAs/lncRNAs in serum of glioma patients. For example, exosomal circ_0072083 was reported to contribute to TMZ resistance in glioma *via* modulating exosomal miR-1252-5p-mediated nanog homeobox (NANOG) degradation. Moreover, exosomal circ_0072083 could serve as an independent diagnostic target for gliomas with an AUC of 0.85, and patients with high expression of exosomal circ_0072083 displayed poorer OS (123). Also, Yin’s team found that exosomal circMMP1 in serum facilitated the progression of glioma *via* functioning as a competitive endogenous RNA (ceRNA) of miR-433 (124). Furthermore, exosomal lncSBF2-AS1 transferred from TMZ-resistant GBM cells to chemoresponsive GBM cells could function as a ceRNAs for miR-151a-3p, contributing to the TMZ resistance (125). Consistently, based on the findings from Hao’ group, lncRNA PTENP1 could be packaged into exosomes of human umbilical cord mesenchymal stem cells and transferred into U87 glioma cells, and subsequently impaired the cell growth *via* suppressing the expression of miR-10a-5p (126). All these studies have demonstrated that exosomal ncRNAs could be served as the promising diagnostic/prognostic markers and therapeutic targets.

To sum up, the multiple exosomal ncRNAs, miRNAs in particular, from serum, plasma, CSF and other body fluids will promote the broad application of exosome-based liquid biopsy strategies in the early diagnosis, prognosis and therapeutic response prediction of gliomas.

### The Potential Application of Exosomal miRNAs in Anti-Glioma Treatment

Given the pivotal biological meanings of exosomal miRNAs in glioma, approaches that specifically target exosomes or exosomal cargoes including miRNAs, are emerging as promising therapies for gliomas. Among the potential therapeutic applications of exosomes, significant attention has been dedicated to applying these vesicles as nanocarriers to deliver small molecules, proteins and nucleic acids such as miRNAs (127). Despite the development of novel methods to engineer exosomal cargoes in producer cells, low yields of exosomes impede the widespread application of exosome-based therapies. Thereupon, some studies have been established to regulate exosomes production to treat cancer. For example, Li et al. confirmed that two intriguing compounds termed MOPIPP and vacuolin-1 could facilitate the vacuolization of endosomal compartments and disrupt the trafficking of late endosomes to lysosomes without exhibiting significant cytotoxicity. Thus exosomes production in GBM cells was enhanced, whereas selected miRNAs carried by the exosomes were identified to show qualitative similarity to those in untreated cells, suggesting that MOPIPP and vacuolin-1 could help develop exosome-based anti-cancer therapeutics (128). However, the potential action mechanism of these compounds should be further explored.

MiRNAs may be a novel antineoplastic agent as they can modulate the posttranscriptional expression of target genes (129–132). Restitution of several downregulated tumor-suppressor miRNAs could repress the growth of GBMs, indicating that certain miRNAs could be used for anti-glioma therapeutics (133). Currently, it seems unsuitable to use available vehicles to deliver miRNAs containing liposomes and viral vectors because of their low efficiency and safety (134). As nature miRNAs carriers, exosomes may be employed to provide tumor-suppressor miRNAs to mediate tumor growth suppression and realize personalized treatment (**Table 3**). For example, Fareh et al. engineered patients’ derived GSCs to stably and continuously express the miR-302-367 cluster, and a large amount of tumor-suppressor miRNA was enclosed in exosomes which were absorbed by neighboring GSC. Thus, its targets containing Cyclin D1, Cyclin A, E2F transcription factor 1 (E2F1) and C-X-C motif chemokine receptor 4 (CXCR4) pathway were repressed, so did the proliferation and tumorigenicity of GSCs (135). Emerging studies have proved that decoy or sponge-like constructs could be employed for miRNA inhibition. They have potential therapeutic benefits by binding complementary miRNA(s) or seed sequences, which can impede the crosslink between miRNAs and their targets (147–149). Monfared and colleagues tried to down-regulated the expression of miR-21 in GBM cell lines, U87-MG and C6, through utilizing engineered exosomes packed with a miR-21-sponge construct. They found that cells treated with miR-21-sponge exosomes exhibited a decline in proliferation and an elevation in apoptotic rates (136). Thereupon, exosomes can be used as a promising therapeutic delivery vehicle in glioma treatment.

**Table 3:** Identification of exosomal miRNAs as potential anti-glioma therapeutics.

Exosomal miRNAs	Donor cells/recipient cells	Targets	Anti-glioma effects	References
miR-302-367	GSC^xvii^/GSC	Cyclin D1, CyclinA, E2F1, CXCR4 pathway	Suppressing proliferation	(135)
a miR-21-sponge construct	HEK-293T/U87-MG	PDCD4 and RECK	Suppressing proliferation	(136)
			Increasing apoptosis	
miR-124a	MSCs^xviii^/ GSCs	(FOX)A2	Suppressing proliferation	(137)
miR-512-5p	BMSCs^xix^/GBM cells	JAG1	Suppressing proliferation	(138)
			Inducing cell cycle arrest	
miR-29a-3p	MSC/glioma cells	ROBO1	Suppressing migration and VM	(139)
miR-7	MSCs/GBM^xx^ cells	XIAP	Enhancing TRAIL sensitivity	(140)
			Increasing apoptosis and suppressing tumor growth	
miR-146b	Marrow stromal cells/ glioma cells	EGFR, NF-κB and SMAD4	Suppressing growth, invasion and migration	(141)
miR-133b	MSC/glioma cells	EZH2	Suppressing proliferation, invasion and migration	(142)
miR-584-5p	MSC/glioma cells	CYP2J2	Suppressing proliferation and migration	(143)
		MMP-2	Suppressing metastasis	
		Bcl-2 and Bax	Induce glioma cells apoptosis	
miR-375	Marrow stromal cells/ glioma cells	SLC31A1	Suppressing proliferation, migration, invasion	(144)
			Increasing apoptosis	
miR-199a	MSC/glioma cells	AGAP2	Suppressing proliferation, invasion and migration	(145)
			Enhancing chemosensitivity to TMZ	
miR-124	WJ-MSC^xxi^/GBM cells	CDK6	Suppressing proliferation	(146)
		IQGAP1, LAMC1, ITGB1	Suppressing migration	
		R-Ras and N-Ras	Increasing chemosensitivity to TMZ	

^xvii^glioma stem-like cells.

^xviii^mesenchymal stem cells.

^xix^bone mesenchymal stem cells.

^xx^glioblastoma.

^xxi^Wharton’s jelly MSCs.

Recently, accumulative evidence has highlighted that mesenchymal stem cells (MSCs), as a novel approach, can overcome the demerits of previous therapies for gliomas (150, 151). MSCs can pass through the BBB and possess inherent tropism towards tumors and low immunogenicity (152). Additionally, MSCs may exert their therapeutic capacity *via* producing and secreting useful exosomes in glioma treatment (153). Several investigations have suggested the delivery of tumor-suppressive miRNAs to cancers through MSC-derived exosomes (154, 155). Studies on glioma have identified that exosomes displayed anti-glioma effects when tumor-suppressive miRNAs expression was elevated in the exosomes-donating MSCs, which revealed the considerable potential for the application of MSC-derived exosomes in treating gliomas. To the end, Lang and colleagues found that MSCs could be exploited as natural biofactories to produce exosomes carrying supraphysiological levels of miR-124a, an effective anti-glioma agent Exo-miR124a exosomes resulted in significantly repressing the viability and clonogenicity of GSCs. When systemically administered, the mice bearing intracranial GSCs xenografts were cured. Mechanistic studies proved that miR-124a acted through silencing Forkhead box (FOX)A2, contributing to abnormal intracellular lipid accumulation (137). Moreover, Yan and co-workers found that miR-512-5p was poorly expressed in GBM tissues and cells, and exosomes derived from miR-512-5p-transfected BMSCs were internalized by U87 cells, thus interfering with GBM cell proliferation and inducing cell cycle arrest through negatively regulating Jagged 1 (JAG1) (138). Furthermore, miR-29a-3p, a tumor suppressor in diverse malignant tumors, was demonstrated to directly target roundabout guidance receptor 1 (ROBO1), thus mitigating vasculogenic mimicry (VM) formation in gliomas. MSCs could be engineered to produce miR-29a-3p-overexpressing exosomes, and treatment with these exosomes restrained migration and VM formation, thereby suppressing the growth of glioma *in vitro* and *in vivo* (139). VM, a kind of alternative microvascular circulation bypassing the canonical VEGF-driven angiogenesis, shows resistance to anti-angiogenesis therapy (156). Tumor necrosis factor-related apoptosis-inducing ligand (TRAIL) has strong potential to kill cancerous cells by inducing apoptosis, with minimal effect on normal cells (157). Zhang et al. identified that miR-7 could sensitize GBM cells to TRAIL-induced apoptosis and reinforced expression of miR-7 in TRAIL-overexpressed MSCs, increased apoptosis and repressed tumor growth in an exosomes-dependent fashion (140). Additionally, based on the report of Katakowsk’s group, the delivery of exosomes derived from MSCs directly by intratumoral injections prominently decreased the growth of glioma xenograft in the rat brain with the inclusion of miR-146b. The anti-tumor effect of MSCs-derived exosomes was underpinned by concurrent inhibition of factors including EGFR, NF-kappaB and mothers against decapentaplegic homolog 4 (SMAD4) (141). It was also found that MSCs-derived exosomes transmitting miR-133b into glioma cells could inhibit EZH2 expression by disrupting the Wnt/β-catenin signaling pathway, thereupon repressing proliferation, invasion and migration of glioma cells (142). Moreover, transferring miR-584-5p through exosomes derived from MSCs could suppress the proliferation and migration of U87 cells *via* decreasing levels of cytochrome P450 family 2 subfamily J member 2 (CYP2J2). This move could also repress glioma metastasis through reducing expression of Matrix metalloproteinase-2 (MMP-2), and induce carcinoma cells apoptosis *via* decreasing levels of B-cell lymphoma-2 (BCL-2), an anti-apoptotic protein, and increasing expression levels of BCL-2 associated X (BAX), a pro-apoptotic protein (143). Exosomal miR-375 from human marrow stromal cells (hMSCs) resulted in suppressed cell proliferation, invasion and migration while promoted apoptosis through solute carrier family 31 member 1 (SLC31A1) inhibition (144). Similarly, miR-199a, when delivered by MSCs *via* the exosomes, could negatively regulate ArfGAP with GTPase domain, ankyrin repeat and PH domain 2 (AGAP2), thereby repressing the proliferation, invasion and migration of glioma cells *in vivo*, and ameliorating the chemosensitivity to TMZ and suppressing the tumor growth *in vivo* (145).

Until now, Studies of MSCs have focused primarily on BMSCs. However, the derivation of stem cells from Wharton’s jelly (WJ) in the human umbilical cord also possesses various advantages. Compared to other sources of MSCs, Wharton’s jelly MSCs (WJ-MSCs) display greater expansion capacity, a higher rate of proliferation, reinforced neurotrophic factors and a stronger potency to default neuronal lineage differentiation. Although these cells are characterized by exceptionally low immunogenicity, they have high neuroprotective potential and appear as a superior source for the application of MSCs without ethical limitations (158–160). Sharif et al. confirmed that WJ-MSCs could efficiently deliver exogenous miR-124 to U87 GBM cells *via* exosomes, ameliorating GBM cells’ chemosensitivity to TMZ and decreasing proliferation and migration of GBM cells. Mechanically, mitigated expression of cyclin-dependent kinase 6 (CDK6) by the delivered miR-124 diminished the proliferation of GBM cells. Besides, the migration of GBM cells was attributed to the inhibition of IQ motif containing GTPase activating protein 1 ((IQGAP1), laminin c1 (LAMC1) and integrin b1 (ITGB1). At the same time, miR-124 also governed the chemosensitivity of GBM cells by targeting R-Ras and N-Ras (146). These studies suggest that MSCs could naturally package anti-tumor miRNAs into exosomes, illustrating their considerable potential for therapeutic applications. Nevertheless, many in-depth studies are required before these therapeutic strategies are available for clinical usage.

## Databases Associated With Exosomal miRNAs

Exosomal miRNAs are key factors in causing various types of cancers, including gliomas. Therefore, it has attracted attention. In recent years, a series of databases linked to miRNAs have been developed, offering information concerning miRNAs and their targets. Deep base database can be used for the comprehensive annotation and mining of small RNAs containing miRNAs, lncRNAs and circRNAs sequencing from transcriptomic data. Thus, the expression of diverse non-coding RNAs and downloading data can be viewed (161). TargetScan (162) and PicTar (163) offer miRNA target predictions by relying on the complementarity between sequence and target sites, emphasizing the perfect base pairing within the seed region and sequence conservation. miRbase is a primary public repository where published miRNA sequences and annotation are archived, and a target gene prediction service is also provided (164). MiRDB, an online platform for miRNA target prediction and functional annotations, uses common features related to miRNA binding and target down-regulation, enabling the identification and prediction of miRNA targets with machine-learning methods (165). MicroRNA appears as a comprehensive resource of miRNA target predictions and expression profiles. It is implemented by miRanda algorithm, which combines current biological knowledge regarding target rules and the application of an up-to-date compendium of mammalian miRNAs (166). Hsu and colleagues developed an integrated resource, miRNAMap 2.0, which was helpful to elucidate the miRNA/target association, especially for humans, by employing three computational tools including Miranda, RNAhybrid and TargetScan to verify miRNA targets within 3’-UTR of the gene and known miRNA targets (167).

Moreover, some databases presented evidence for experimentally verifying miRNAs and their target genes. To this end, DIANA-TarBase, as a manually curated database, contains experimentally verified miRNA-target interactions, with detailed information concerning meta-data, experimental methodologies and conditions (168). MiRWalk offers predicted and validated information about miRNA-targe interaction and allows researchers to verify novel miRNA targets in the 3′-UTR and other regions of the known genes (169). Yang et al. introduced a novel database, namely starBase, which includes high-throughput sequencing data derived from 21 Argonaute CLIP-Seq and 10 Degradome-Seq experiments performed in 6 organisms. This database can contribute to the comprehensive investigation of miRNA-target interaction maps (170). miRecords, an integrated resource providing animal miRNA-target gene prediction, hosts a large and high-quality database of experimental tested miRNA-target interactions, which emphasizes the systematic and structured experimental documentation for each interaction (171).

## Conclusion and Perspective

Exosomes, as a sub-group of EVs, serve as major conduits for cell-to-cell communication. Intercellular signal transduction *via* shuttling molecular cargo mediates the physiologic and pathophysiologic processes of diverse recipient cells. According to current findings, miRNAs can be selectively absorbed into exosomes and serve essential roles in the proliferation, invasion and migration, angiogenesis, therapeutic resistance and immune suppression of gliomas. Furthermore, the fact that exosomes carry many bioactive substances and can easily pass-through biological barriers, including BBB. Thus, they have great potential for clinical application, covering diagnosis, prognosis, and therapy.

Although the outlooks of exosomal miRNAs in the clinical application are broad, there are some challenges. For instance, standard technology is a must for the isolation and purification of exosomes. Exosomes in various body fluids often represent a heterogeneous group of vesicles, the origin of which is unknown. Consequently, the identification and isolation of the exosomes associated with specific tumors are extremely critical. However, this could be difficult to realize, as multiple approaches for isolation have their own merits and demerits. Additionally, the maximum use, normalization and quantitation of exosomes are still problems, as the number of genetic materials in biofluid exosomes is limited. More detailed investigations are required to broaden our current knowledge about exosome biogenesis, secretion and uptake, and specific sorting mechanisms of exosomal miRNAs. If we work out mechanisms underlying these complex processes, we may readily manipulate the exosomes, pack miRNAs, proteins or DNA of interest, and target exosomes to specific cell types or tissues, thus malignant glioma patients can be effectively treated. Finally, large-scale prospective studies are needed to obtain results with high reproducibility and substantiate the efficacy and safety of exosomal biomarkers management in patients. In conclusion, with the continuous understanding of these unknown substances, exosomes will exhibit increasing application value in clinical diagnosis, prognosis and therapy of cancers.

## Author Contributions

Conception and design: JP, QL, ZX, YY, and MZ. Writing, review, and/or revision of the manuscript: QL, YC, BP, JL, and WZ. Administrative, technical, or material support: FK, QH, and MZ. All authors approved final version of manuscript.

## Funding

This study is supported by grants from the Science and Technology Innovation Program of Hunan Province (2021RC3029), the China Postdoctoral Science Foundation (2021T140754, 2020M672521), the Natural Science Foundation of Hunan Province (2020JJ5934), and the Postdoctoral Science Foundation of Central South University (248485).

## Conflict of Interest

The authors declare that the research was conducted in the absence of any commercial or financial relationships that could be construed as a potential conflict of interest.

## Publisher’s Note

All claims expressed in this article are solely those of the authors and do not necessarily represent those of their affiliated organizations, or those of the publisher, the editors and the reviewers. Any product that may be evaluated in this article, or claim that may be made by its manufacturer, is not guaranteed or endorsed by the publisher.
